# Production of a Soluble Recombinant Antibody Fragment against MMP9 Using *Escherichia coli*

**DOI:** 10.3390/medicina57090981

**Published:** 2021-09-18

**Authors:** Chang-Hun Yeom, Hee-Jin Jeong

**Affiliations:** Department of Biological and Chemical Engineering, Hongik University, Sejong 30016, Korea; ysksun9@gmail.com

**Keywords:** matrix metalloproteinase, recombinant antibody, *Escherichia coli*, soluble expression

## Abstract

Matrix metalloproteinase 9 (MMP9) is involved in several aspects of the pathology of cancer, including invasion, metastasis, and angiogenesis. In this study, we expressed a recombinant scFv-type anti-MMP9 antibody in soluble form using *Escherichia coli*, purified it, and confirmed its antigen-binding ability. The convenient, rapid, inexpressive system used in this study for producing recombinant antibody fragments needs only five days, and thus can be used for the efficient production of scFv against MMP9, which can be used in a range of applications and industrial fields, including diagnosis and treatment of inflammatory and cancer-related diseases.

## 1. Introduction

Matrix metalloproteinases (MMPs) belong to a neutral protease family and are involved in the pathogenesis of different forms of cancers and arthritis [1]. MMP9 is an enzyme that is produced by cells in the vascular wall, vascular smooth muscle cells, and inflammatory immune cells such as neutrophils, monocytes, and endothelial cells [2,3]. MMP9 is structurally divided into three domains: a pro-peptide, a catalytic domain that has a zinc ion binding site, and a haemopexin-like domain 3 [4]. MMP9 is secreted in a latent form, as a pro-enzyme, and exists in an inactive state by combination with a zinc ion, with the help of the cysteine residue of the pro-peptide. The pro-peptide is released, and MMP9 is activated by MMP3 [5,6]. Activated MMP9 is involved in tissue remodeling in physiological and pathological processes such as morphogenesis, tissue repair, and metastasis, as well as in the destruction of matrix components other than collagen, elastin, and gelatin, which leads to the destruction of inflammatory tissues [7,8]. Due to its involvement in a variety of diseases, MMP9 has been of considerable interest in biomedical and pharmaceutical research. The overexpression of MMP9 is a potential biomarker in cancer, because MMP9 promotes the development and progression of cancers, is associated with tumor growth, and mediates tumor invasion, metastasis, and the tumor microenvironment [9,10,11,12]. Several studies have investigated MMP9 as a target molecule for the diagnosis and treatment of inflammatory conditions and cancer. In preclinical investigations, inhibition of MMP9 reduced disease severity in a mouse model of ulcerative colitis, and decreased tumor growth and metastasis in a surgical orthotopic xenograft model of colorectal carcinoma. The inhibition of either tumor- or stroma-derived MMP9 has been shown to be sufficient for reducing primary tumor growth [13]. 

Research into the development of a selective monoclonal antibody that can be used as an inhibitor of MMP9 is currently underway. As an antibody has high selectivity for its antigen, highly specific detection and recognition of target molecules is possible using antibodies. There is considerable interest in the production of a recombinant antibody that expresses the nucleotide sequence of a monoclonal antibody, generated by immunizing an animal with an antigen and then recombining the sequences for production in *Escherichia coli* (*E. coli*) or animal cells. The use of recombinant antibodies has several advantages: the production period is short, no animals are used, and large amounts of antibody can be produced at low cost. When using *E. coli* as a host, the production time is shorter, the risk of contamination is lower, and the media is less expensive than when animal cells such as HEK or CHO cells are used. In case of *E. coli*-based antibody generation, although the expression of full-sized antibody is possible, the production of antibody fragments in the form of antigen-binding fragments (Fabs) or single-chain variable fragments (scFvs) is predominant. An scFv is the smallest antibody fragment containing whole complementarity-determining regions that can perform a functional role in antigen-binding, and can be produced with a high yield at a low cost. scFvs are approximately 15 kDa in size—smaller than Fabs, which are approximately 30 kDa, or full-sized antibodies of approximately 150 kDa. Because of their small size, scFvs can relatively easily penetrate dense tumor cells or whole blood, allowing the elimination of pre-treatment steps for detecting target molecules [14,15]. Thus, several studies are being conducted into the use of scFvs for diagnosis, treatment, and therapy [16]. Moreover, since the hinge region and Fc antibody fragment contain glycosylation sites, glycosylation is not formed in scFv and Fab [17]. Due to the absence of glycosylation in scFv, *E. coli*-based expression has been focused on as a proper system for scFv production, even though glycosylation is lacking in *E. coli* [17,18,19]. Commercial enzymes, such as papain for obtaining Fab and pepsin for obtaining F(ab′)_2_, to digest full-sized antibodies are widely available, and can digest anti-MMP9 antibodies to obtain those fragments [20]. However, the efficiency of enzymatic digestion varies by enzyme activity, depending on several reaction parameters including pH, enzyme concentration, reaction temperature, and reaction time [21]. Therefore, optimization of these parameters is required to increase the yield and efficiency of fragmentation; in particular, as enzymes show high activity at 37 °C whereas antibodies have high stability at 4 °C, it is crucial to control the reaction temperature to maintain the structure of antibodies and prevent aggregation or denaturation of antibodies during enzymatic digestion. Conversely, recombinant antibodies are generated from synthetic genes. Once the sequence of variable domains of an antibody is cloned, it is possible for it to be modified into several types of antibody fragments, including Fab, F(ab′)_2_, scFv, (scFv)_2_, and dsFv; this indicates a higher structural diversity of recombinant antibody fragments than enzymatically digested antibody fragments, as enzyme digestion-based methods can only produce Fab and F(ab′)_2_ [20].

Recently, anti-MMP9 Fab has been generated by digesting a humanized monoclonal anti-MMP9 antibody, GS-5745, with an enzyme; its structure, function, and positive effects in the treatment of ulcerative colitis and gastric cancer was shown [22]. The therapeutic promise of GS-5745 led to clinical trials. GS-5745 was found to be a potent and highly selective inhibitor of MMP9, without side effects [13]. A study of GS-5745 combined with mFOLFOX6 demonstrated its efficacy, without added toxicity, in a clinical study of gastric and gastroesophageal junction adenocarcinoma [23,24].

In this study, we expressed an anti-MMP9 antibody in scFv form using *E. coli*, through the sequence of the variable domain of GS-5745. After codon optimization of the sequences, we succeeded in producing soluble expression of the scFv in *E. coli*, and confirmed the activity of the purified scFv using indirect enzyme-linked immunosorbent assay (ELISA). 

## 2. Materials and Methods

### 2.1. Materials

KOD-plus Neo kit was obtained from Toyobo (Osaka, Japan). Infusion enzyme was obtained from TaKaRa (Tokyo, Japan). *E. coli* SHuffle T7 Express lysY were obtained from New England Biolabs Korea (Seoul, Korea). A plasmid miniprep kit and oligonucleotides were obtained from Bionics (Daejeon, Korea). His Sepharose Ni was obtained from GE healthcare (Piscataway, NJ, USA). The Nanosep Centrifugal-3 k Ultrafiltration Device was obtained from Pall Corporation (Ann Arbor, MI, USA). Maxi plates were obtained from SPL Life Sciences (Gyeonggi-do, Korea). Anti-DYKDDDDK-HRP conjugate antibody was obtained from (Biolegend, CA, USA) and 3,3′,5,5′-Tetramethylbenzidine (TMB) was obtained from Sigma (St. Louis, MO, USA). Purified MMP9 protein was obtained from Sino (Beijing, China). Purified catalytic domain of MMP9 was obtained from Abcam (Cambridge, United Kingdom). Other chemicals and reagents, unless otherwise indicated, were from Sigma (Seoul, Korea). 

### 2.2. Construction of Anti-MMP9-scFv Gene

To construct pSQ:aMMP9scFv, the anti-MMP Fab coding gene (PDB: 5th9) [22] with both an N-terminal Cys-tag and C-terminal His- and Flag- tags was chemically synthesized and amplified by polymerase chain reaction (PCR) using primers NCSNE Fw (5′-cgaagtaaactgctctaatgag-3′) and GGGSH Rv (5′-atgatgatgagaacccccccc-3′), and KOD-plus Neo DNA polymerase. The product was ligated to pSrtCys vector, which was amplified by PCR using pSQ vector [25], and Vec Fw (5′-ggggggggttctcatcatca-3′) and Vec Rv (5′-ctcattagagcagtttacttcgatttgagc-3′) as primers, using In-Fusion enzyme. The PCR mixtures contained 5 μL of 10x buffer, 5 μL of 2 mM dNTPs, 3 μL of 25 mM MgSO_4_, 1 μL of 10 μM primer pairs, template DNA 50 ng, and enzyme 1 U, up to a volume of 50 μL with distilled water. Amplification of insert DNA was performed under the following conditions: 94 °C for 2 min; 35 cycles of 98 °C for 10 s, 54 °C for 30 s, and 68 °C for 30 s. Amplification of vector DNA was performed the following conditions: 94 °C for 2 min; 35 cycles of 98 °C for 10 s, 49 °C for 30 s, and 68 °C for 180 s. The obtained plasmids were prepared using the plasmid miniprep system, and the entire coding-region sequences were confirmed by sequencing. 

### 2.3. Expression and Purification of Protein

SHuffle T7 Express lysY cells were transformed with pSQ:aMMP9scFv and cultured at 37 °C for 16 h in LBA medium (LB medium containing 100 μg/mL ampicillin) and 1.5% agar. Single colonies were picked and grown at 37 °C in 4 mL of LBA medium overnight, from which 1 mL was used to inoculate 100 mL of LBA medium. The cells were cultured at 37 °C until an OD_600_ of 0.6, after which 0.4 mM isopropylthio-β-galactopyranoside (IPTG) was added. The solution was incubated for an additional 16 h at 16 °C, followed by centrifugation (4000× *g*, 30 min, 4 °C). The pellet was resuspended using 8 mL of binding buffer (50 mM phosphate (pH 7.4), 0.3 M sodium chloride (NaCl), and 10 mM imidazole) and sonicated. After centrifugation (4000× *g*, 30 min, 4 °C), the supernatant was mixed with 200 μL of Ni Sepharose resin on a rotating wheel for 1 h at room temperature. The beads were washed three times with 10 mL of washing buffer (50 mM phosphate (pH 7.4), 0.3 M NaCl, and 20 mM imidazole). After adding 4 mL of eluting buffer (50 mM phosphate (pH 7.4), 0.3 M NaCl, and 300 mM imidazole), the mixture was incubated for 1 h at room temperature. The eluent was subjected to an ultrafiltration device (MWCO 3 k), equilibrated with PBS (10 mM phosphate, 137 mM NaCl, 2.7 mM potassium chloride (pH 7.4)), and concentrated to 250 μL. Protein expression and purification were confirmed by SDS-PAGE analysis, and protein concentration was determined using ImageJ software (National Institute of Health, Bethesda, MD, USA) with a varied concentration of bovine serum albumin (BSA) as a standard.

### 2.4. Enzyme-Linked Immunosorbent Assay

The antigen-binding activity of anti-MMP9 scFv was confirmed by indirect ELISA. A varied amount of MMP9 or catalytic domain of MMP9 in 100 μL PBS was immobilized on each well of a 96-well plate for 8 h at 4 °C. The well was blocked with 350 μL of 3% BSA in TBST (TBS buffer containing 0.1% Tween20) for 2 h at room temperature and washed three times with TBST. Subsequently, 100 μL of 1 μg/mL anti-MMP9 scFv in TBSTB (TBST containing 0.1% BSA) was added and incubated for 30 min at room temperature. The well was washed three times with TBST and incubated with 100 μL/well of 10,000-fold diluted HRP-conjugated anti-Flag antibody in PBS for 1 h at room temperature. The well was washed three times with TBST and developed with 50 μL/well of TMBZ solution. After incubation for 10 min, the reaction was stopped by adding 50 μL/well of 10% sulfuric acid, and the absorbance was read at 450 nm using a microplate reader. As a control, PBS was employed instead of MMP9, and the same procedure was performed.

## 3. Results

### 3.1. Construction of an Anti-MMP9 scFv Coding Gene

Marshall et al. expressed MMP9 protein by transfecting the full-length cDNA of MMP9 into HEK293 cells. The purified MMP9 was injected into mice, and a library of antibodies against MMP9 was generated via a hybridoma system. The monoclonal antibody that had highest antigen-binding efficiency to MMP9, AB0041, was selected. The variable domains and kappa chains of the AB0041 were humanized to generate a clinical anti-MMP9 antibody, which was named GS-5745. GS-5745 showed potency and selectivity equivalent to that of AB0041, and inhibited MMP9 [13].

We performed codon optimization of the VH and VL sequences of GS-5745 to generate an anti-MMP9 scFv using *E. coli* (Table 1). We genetically synthesized the anti-MMP9 scFv gene, which was composed of a VH-linker-VL (VH and VL, linked by a GGGS peptide linker), and inserted the gene into a pSrtCys vector—a modified pSQ vector [25] in which a GGGGG-tag was located between the start codon and the Cys-tag (described elsewhere). We also added a His-tag at the C-terminal of scFv for protein purification, followed by a Flag-tag. As the recombinant MMP9 which we used as an antigen for ELISA had a His-tag for its expression in *E. coli* and for its His-tag-based purification, we were not able to use HRP-conjugated anti-His-tag antibody as a secondary antibody for ELISA. Therefore, we added a Flag-tag next to the His-tag, and used HRP-conjugated anti-Flag antibody as the secondary antibody.

### 3.2. Expression of Soluble Anti-MMP9 scFv

**Figure 1:**
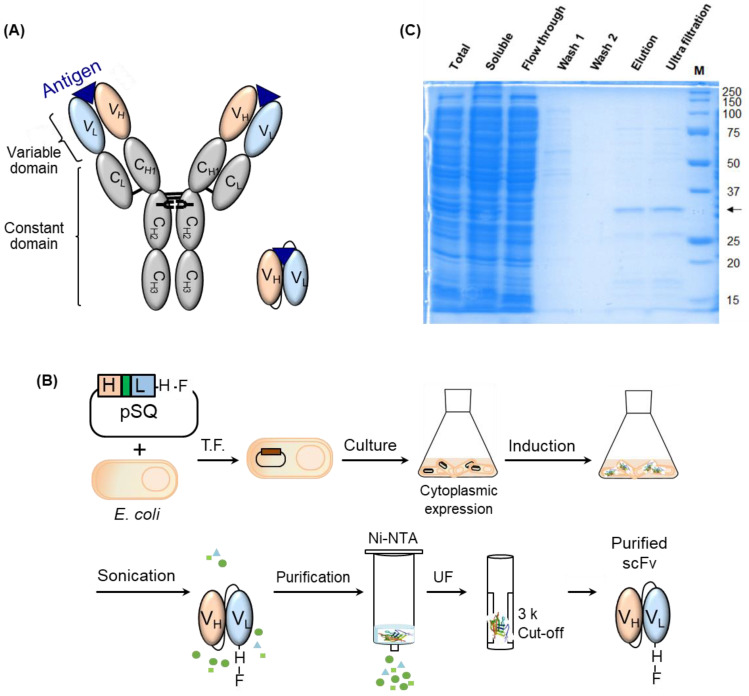
(**A**) Schematic representation of full-sized antibody and scFv, (**B**) Schematic representation of all steps for expressing and purifying anti-MMP9 scFv; H, F, T.F., and UF indicate His-tag, Flag-tag, transformation, and ultra-filtration, respectively, (**C**) SDS-PAGE analysis of expressed or purified anti-MMP9 scFv. The arrow indicates the band of target protein.

We expressed anti-MMP9 scFv using *E. coli* and purified the proteins in the cytosol by immobilized metal affinity chromatography (Figure 1A,B). At that time, we optimized the culture medium, induction temperature, induction time, IPTG concentration, imidazole concentration in washing buffer, and the volume of washing buffer, to improve expression yield and purity (Figure S1). Finally, we confirmed that 102 μg of purified scFv fragment with the expected size of 29.2 kDa was obtained in soluble form from 100 mL shake-flask culture when using TB medium, induction with 0.4 mM IPTG for 16 h at 16 °C, and 10 mM imidazole-containing washing buffer (Figure 1C). We could show that the extra bands were the proteins extracted from *E. coli* by comparing the expressed scFv bands to the ones from the sample with no addition of IPTG during an induction step. As the extra proteins were nonspecifically bound to the His-tag beads and were not fully eliminated after washing, they remained in the sample. Although some nonspecific bands were observed on the SDS-PAGE gel, we did not perform further additional purification to prevent the loss of the target protein and maintain the production yield. As nonspecific proteins have no binding activity to the target antigen, they could be washed during the ELISA, resulting in no effect on the response of the target antibody. We determined the concentration of target protein via densitometric analysis using ImageJ software instead of the absorbance-based method or Bradford assay. Therefore, not the concentration of whole proteins, including nonspecific proteins in the sample, but the exact concentration of target proteins was calculated.

Recombinant protein expression requires high expression yields, and *E. coli* is suitable for this purpose because it is inexpensive, has a fast growth rate, is simple to use, and a large number of compatible molecular tools are available [26,27]. However, in spite of all these qualities, obtaining high yields of soluble protein is challenging, and expressed protein often forms insoluble aggregations known as inclusion bodies. These aggregated proteins are in general misfolded, and thus biologically inactive and nonfunctional [28]. To overcome this issue, several protein refolding methods have been adapted. However, the major obstacles to this production process are the poor recovery yields, the requirement for optimization of refolding conditions, and the possibility that re-solubilization procedures could affect the integrity of refolded proteins. The purification of soluble protein is less expensive and less time consuming than refolding and purification from inclusion bodies [29].

### 3.3. Antigen-Binding Efficiency of Anti-MMP9 scFv

**Figure 2:**
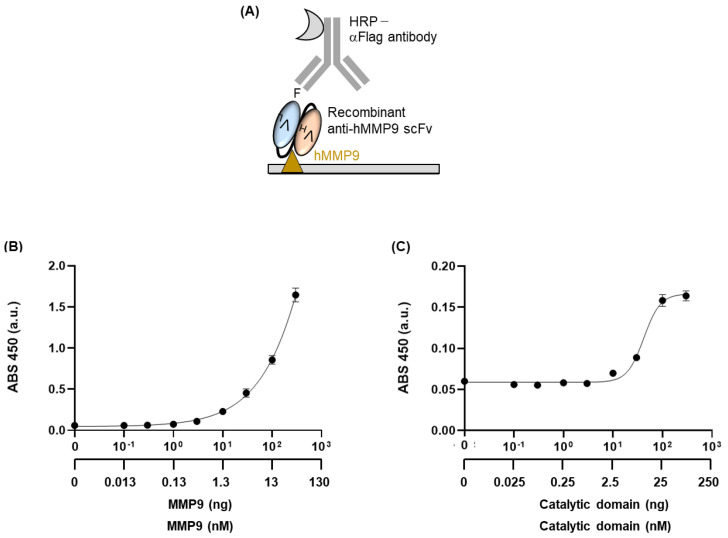
(**A**) Schematic representation of indirect ELISA for confirming the antigen-binding efficiency of anti-MMP9 scFv, (**B**) Titration curve of ELISA with MMP9 as an antigen, (**C**) Titration curve of ELISA with the catalytic domain of MMP9 as an antigen. Error bars represent ±1 SD (*n* = 3).

We examined the antigen-binding activity of purified scFv using ELISA. We seeded several concentrations of commercially available recombinant MMP9 protein onto a 96-well plate and blocked the wells. Afterwards, we added scFv as a primary antibody, followed by HRP-conjugated anti-Flag antibody as a secondary antibody (Figure 2A). The signals were increased in the presence of antigens, whereas relatively lower signals were observed from the wells without antigens. The titers against MMP9 were shown to be antigen concentration-dependent, indicating that scFv has great binding properties against its target (Figure 2B). Although the titration curve was not saturated at the maximum antigen concentration of this ELISA system (300 ng), it could reach a plateau if the antigen-binding efficiency of the antibody was further increased or nonspecific binding during the ELISA procedure was eliminated.

There is no published limit of detection (LOD) value for the original anti-MMP9 antibody, GS-5745, but its EC50 value—the concentration of a drug that gives half-maximal response—was determined as 0.218 ± 0.040 nM [13]. Although the exact comparison of binding abilities between scFv and original full-sized antibody was unavailable due to differences in the reagents used for each ELISA system, the EC50 value of the original antibody was lower than the scFv. This might be due to structural differences, such as the presence or absence of a peptide linker between the VH and VL domains, or production method differences. Although, the binding ability of the newly generated antibody was lower than the referenced full-sized antibody, which was produced via hybridoma cell culture—requiring the fusion of mouse myeloma with B cells obtained from a mouse immunized with hMMP9, followed by humanization, which was time-consuming and labor-intensive. Conversely, the *E. coli*-based production of scFv developed in this study was certainly rapid and simple. Moreover, the LOD value of the scFv (0.454 ng) corresponds to 0.0595 nM, as the molecular size of recombinant hMMP9 used as an antigen was 76.3 kDa, indicating high sensitivity that can detect MMP9 to a nanomolar order.

We confirmed the binding efficiency of this anti-MMP9 scFv to the catalytic domain of MMP9. We used a recombinant catalytic domain of MMP9, which was generated from *E. coli* containing the 113–450 amino acid sequence of whole MMP9. When the zinc ion located between the pro-peptide and the catalytic domain reacts to MMP3, the catalytic domain is separated from MMP9, and the MMP9 without the catalytic domain is activated [22]. Appleby et al. found that GS-5745 binds to Gln108 of MMP9 near the junction between the pro-peptide and the catalytic domain, and that the binding did not interfere directly with the catalytic domain. After the binding of GS-5745 to Gln108, MMP3 cleaves the region between the pro-peptide and the catalytic domain, and activation is inhibited [22]. We obtained a titer that increased in an antigen concentration-dependent manner, with an EC50 value of 41.96 ± 8.74 ng and an LOD of 14.61 ng (Figure 2C). When we compared the LOD value to that of whole MMP9, it was confirmed that the scFv had a higher binding efficiency to whole MMP9 than to the catalytic domain, which is consistent with the proposed lower binding tendency of GS-5745 [13,22].

## 4. Discussion

MMP9 is involved in inflammation and tumor growth, including periodontitis and several types of cancer. In this study, a recombinant scFv-type antibody isolated from anti-MMP9 Fab, which is an enzymatically digested humanized anti-MMP9 antibody, was expressed in soluble form from *E. coli*, and purified. Subsequently, the high antigen-binding ability of the antibody was confirmed using ELISA. The convenient and rapid system established in this study could be used for the efficient production of scFv against MMP9 with high purity and low cost for industrial use. To further improve the purity of the anti-MMP9 scFv for use in clinical trials, size-exclusion chromatography would be useful in removing extra proteins after large-scale production. Unlike enzymatic cleavage methods for producing antibody fragments that are limited to the types of antibody fragments generated and require enzymes, which are of high cost, recombinant antibody production is more suitable as an industrial antibody production platform. This antibody, which binds to MMP9 with high sensitivity, will be valuable in several biomedical fields, including the diagnosis and treatment of inflammatory and cancer-related diseases.

## Figures and Tables

**Table 1:** Sequences of variable domains in anti-MMP9 scFv.

	VH	VL
Nucleotide	caggtgcagctgcaggaaa gcggcccgggcctggtgaaac cgagcgaaaccctgagcctgac ctgcaccgtgagcggctttagcctgctgagctatggcgtgc attgggtgcgccagccgccgggcaaaggcctg gaatggctgggcgtgatttggaccggcgg caccaccaactataacagcgcgctgatgagccgctttaccat tagcaaagatgatagcaaaaacaccgtg tatctgaaaatgaacagcctgaaaaccgaaga taccgcgatttattattgcgcgcgctattattatggcatggatt attggggccagggcaccctggtgaccgtgagcagc	gatattcagatgacccagagcccgag cagcctgagcgcgagcgtgggcgatcgcgtgac cattacctgcaaagcgagccaggatgtgcg caacaccgtggcgtggtatcagcagaaac cgggcaaagcgccgaaactgctgatttatagcag cagctatcgcaacaccggcgtgccg gatcgctttagcggcagcggcagcggcaccgat tttaccctgaccattagcagcctgcaggcggaag atgtggcggtgtattattgccagcagcatta tattaccccgtatacctttggcggcggcac caaagtggaaattaaacgcaccgtg
Amino acid	QVQLQESGPGLVKP SETLSLTCTVSGFSLLSY GVHWVRQPPGKGLEWLGVIWTGGTT NYNSALMSRFTISKDDSKNTVYLKMNSL KTEDTAIYYCARYYYG MDYWGQGTLVTVSS	DIQMTQSPSSLSASVGDRVTITCK ASQDVRNTVAWYQQKPGKAP KLLIYSSSYRNTGVP DRFSGSGSGTDFTLTISSLQAEDVA VYYCQQHYIT PYTFGGGTKVEIKRTV

## Supplementary Materials

The following are available online at https://www.mdpi.com/article/10.3390/medicina57090981/s1, Figure S1: SDS-PAGE analysis of expressed anti-MMP9 scFv under various conditions.
