# Supplementary material for: Production of a Soluble Recombinant Antibody Fragment against MMP9 Using Escherichia coli
Source: Medicina (Kaunas). 2021 Sep 18;57(9):981. doi: 10.3390/medicina57090981 (PMC8468072; doi:10.3390/medicina57090981)
Supplement: Supplementary file 1 [file medicina-57-00981-s001.zip › Figure S1.pdf]

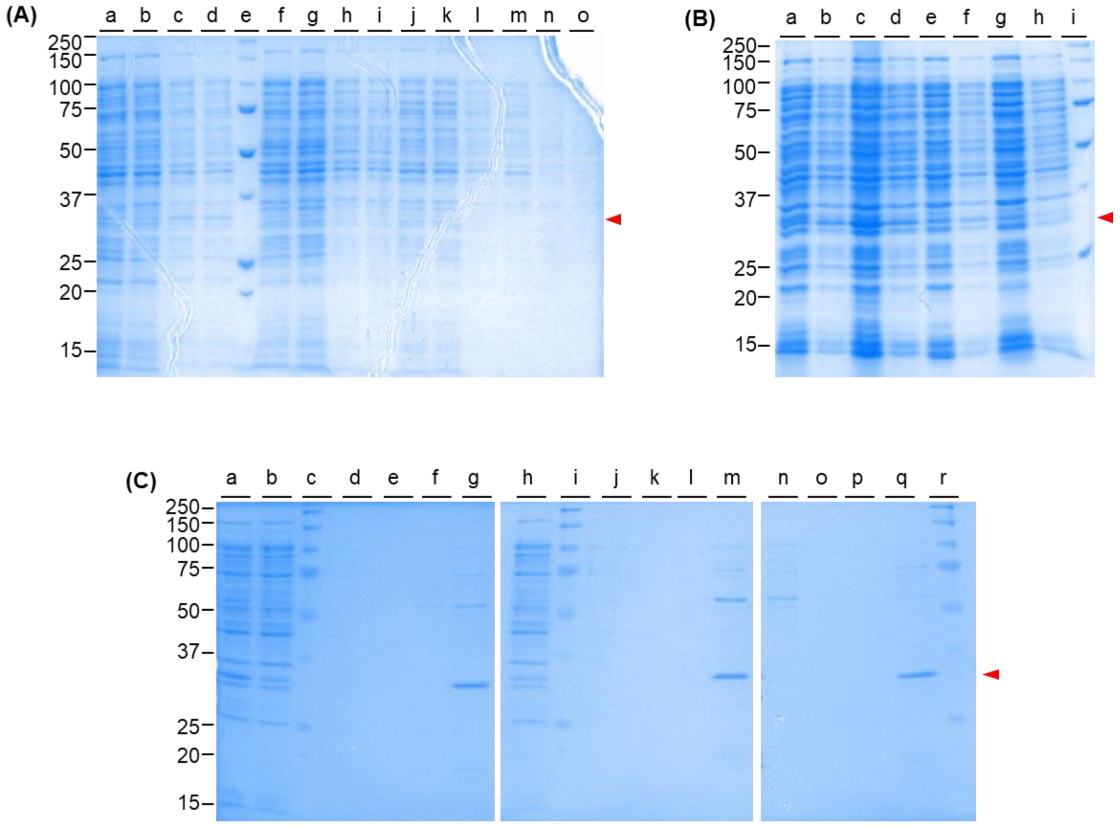

Figure S1. SDS-PAGE analysis of expressed anti-MMP9 scFv under various conditions. (A) Soluble fractions of expressed protein (10 mL scale culture) under different culture medium, induction temperature, induction time, and IPTG concentration. a; TB medium, 16°C, 16 h, 0.4 mM IPTG, b; TB medium, 16°C, 16 h, 1 mM IPTG, c; LB medium, 16°C, 16 h, 0.4 mM IPTG, d; LB medium, 16°C, 16 h, 1 mM IPTG, e; protein ladder, f; TB medium, 30°C, 16 h, 0.4 mM IPTG, g; TB medium, 30°C, 16 h, 1 mM IPTG, h; LB medium, 30°C, 16 h, 0.4 mM IPTG, i; LB medium, 30°C, 16 h, 1 mM IPTG, j; TB medium, 37°C, 4 h, 0.4 mM IPTG, k; TB medium, 37°C, 4 h, 1 mM IPTG, l; LB medium, 37°C, 4 h, 0.4 mM IPTG, m; LB medium, 37°C, 4 h, 1 mM IPTG, n; TB medium, no induction, o; LB medium, no induction. (B) Soluble fractions of expressed protein (50 mL scale culture) under different culture medium and induction temperature with 0.4 mM IPTG and 16 h induction time. a; TB medium, 16°C, total fraction, b; LB medium, 16°C, total fraction, c; TB medium, 30°C, total fraction d; LB medium, 30°C, total fraction, e; TB medium, 16°C, soluble fraction, f; LB medium, 16°C, soluble fraction, g; TB medium, 30°C, soluble fraction, h; LB medium, 30°C, soluble fraction, i; protein ladder. (C) Purified protein under different purification condition, including imidazole concentration in washing buffer and the volume of washing buffer. a; without purification, b; flow-through, c; protein ladder, d; 1st washing using 5 mM imidazole-containing buffer, e; 2nd washing of “d” using 10 mM imidazole-containing buffer, f; 3rd washing of “e” using 20 mM imidazole-containing buffer, g; eluted fraction after “f” using 300 mM imidazole-containing buffer, h; flow-through, i; protein ladder, j; 1st washing using 10 mM imidazole-containing buffer, k; 2nd washing of “j” using 10 mM imidazole-containing buffer, l; 3rd washing of “k” using 10 mM imidazole-containing buffer, m; eluted fraction after “l” using 300 mM imidazole-containing buffer, n; 1st washing using 20 mM imidazole-containing buffer, o; 2nd washing of “n” using 20 mM, p; 3rd washing of “o” using 20 mM imidazole-containing buffer, q; eluted fraction after “p” using 300 mM imidazole-containing buffer; r; protein ladder. The arrow indicates the band of target protein.
